# A framework for modern time geography: emphasizing diverse constraints on accessibility

**DOI:** 10.1007/s10109-023-00404-1

**Published:** 2023-02-16

**Authors:** Somayeh Dodge, Trisalyn A. Nelson

**Affiliations:** Department of Geography, University of California Santa Barbara, Santa Barbara, USA

**Keywords:** Time geography, Mobility analytics, Big data, Social sensing, Geospatial data representativeness, R41 transportation: demand, supply, and congestion, travel time, safety and accidents, transportation noise, C3 multiple or simultaneous equation models, multiple variables, C8 data collection and data estimation methodology, computer programs, C6 mathematical methods, programming models, mathematical and simulation modeling, I14 health and inequality

## Abstract

Time geography is widely used by geographers as a model for understanding accessibility. Recent changes in how access is created, an increasing awareness of the need to better understand individual variability in access, and growing availability of detailed spatial and mobility data have created an opportunity to build more flexible time geography models. Our goal is to outline a research agenda for a modern time geography that allows new modes of access and a variety of data to flexibly represent the complexity of the relationship between time and access. A modern time geography is more able to nuance individual experience and creates a pathway for monitoring progress toward inclusion. We lean on the original work by Hägerstrand and the field of movement GIScience to develop both a framework and research roadmap that, if addressed, can enhance the flexibility of time geography to help ensure time geography will continue as a cornerstone of accessibility research. The proposed framework emphasizes the individual and differentiates access based on how individuals experience *internal*, *external*, and *structural* factors. To enhance nuanced representation of inclusion and exclusion, we propose research needs, focusing efforts on implementing flexible space–time constraints, inclusion of definitive variables, addressing mechanisms for representing and including relative variables, and addressing the need to link between individual and population scales of analysis. The accelerated digitalization of society, including availability of new forms of digital spatial data, combined with a focus on understanding how access varies across race, income, sexual identity, and physical limitations requires new consideration for how we include constraints in our studies of access. It is an exciting era for time geography and there are massive opportunities for all geographers to consider how to incorporate new realities and research priorities into time geography models, which have had a long tradition of supporting theory and implementation of accessibility research.

## Introduction

Hägerstrand’s time geography has been widely used as a generalized model for studying accessibility (Patterson and Farber 2015). Using time geography, access is framed as the ability to utilize a resource or reach an opportunity and is influenced by movement over time. A strength of time geography is that methods, such as the space time prism, can be used to characterize movement patterns based on spatial processes of time and speed, conditioned on the geography of physical and built environments (Song et al. 2016; Tribby et al. 2016). Using this framework, researchers ask questions about how access to services, such as public transportation, varies with community socioeconomics (Páez et al. 2010; Wei et al. 2017), or how access to opportunities varies by individual characteristics, such as gender (Kwan 1999), and conduct analysis to guide improved access to healthcare (Kim et al. 2018).

Recent social and cultural disruptions have added complexity to space–time processes that govern access (Nelson et al. 2022). At the same time, technological advances have created opportunities to enable access by removing the travel need for obtaining goods, services, and necessities (e.g., through eCommerce, Instacart, Telehealth, etc.) and jobs (via remote work), and even for socializing. Nowadays social interactions via the Internet for example on twitter, dating apps, and Metaverse platforms have become ubiquitous. In classic time geography, travel time is a key factor of accessibility. The accelerated digitization of our lives, brought on by COVID-19 lockdowns, has amplified forms of access, and also created barriers to access, that are uncoupled from travel time. For example, a Telehealth appointment provides health care without travel, but it can also prevent receiving health care due to lack of access to digital technologies (Rodriguez et al. 2022). Although access can be achieved without travel, it might differ for different individuals based on who they are and their life circumstances. Health care access is governed more by insurance than by the spatial proximity between healthcare facilities and home, and technology may place new opportunities and barriers to healthcare. For example, online reviews of service providers might also influence the preferences of individuals to access certain services regardless of distance and time. Moreover, even with eliminating the travel need through Telehealth, time still plays a role in health care access, as people cue for appointment times and spend time scheduling and checking-in for appointments. In order for time geography to continue to be a generalized framework for quantifying accessibility, methods are needed to incorporate allocations of time and other individual-specific parameters associated with processes other than movement and to incorporate access that is enabled without travel.

Recently we have witnessed a massive increase in the availability of big data, 80% of which is spatial (Huang and Wang 2020). A particular case of spatial data is mobility datasets, which have increased in availability and use. For example, point of sales data, smartphone GPS generated data, and fitness app data are all being used to understand people movements at an aggregate level (e.g., Nelson et al 2021). Privacy constrains the broad access to granular big mobility datasets. In most cases, granular movement data are held by private companies and researchers have limited access to these data sets, making individual movements primarily available when researchers conduct studies and recruit participants. While individual GPS data are an enhancement compared with traditional travel survey data, and may even be large in terms of file size, they are not big in terms of being generated by a large group of people. There is exciting potential for integrating big mobility data sets with time geography to understand how movement, time, and geography intersect to create individual accessibility. While on the surface it seems we have all the data needed to address these questions, how we integrate the variety of spatial data at both individual and aggregate levels remains a challenge with many details that need to be considered.

Concurrent to the digital revolution, some of which has been fast-tracked by COVID-19, there has been accelerated awareness of how access and time allocation vary for different individuals and communities based on factors, such as race, income, sexual identity, and physical limitations and ability for telecommute (McBride et al. 2020; Su et al. 2021). Current models of access that focus on generic movement patterns and the physical and built environment are missing variation in experience that is social, cultural, and individual. When we generalize individual experience, we run the risk of over representing dominant cultural values and reinforcing systematic racism and other harmful structures. The importance of including varied experience in access modeling is not new. Studies on modeling movement through time geography based on gender (Kwan 1999, 2000) and behavior (Loraamm 2020) have addressed these questions, and have done so by quantifying how space–time use of geography varies for different individuals (McBride et al. 2020). However, we have found few studies that quantify diverse and individual experiences by incorporating data on perception, culture, and social interactions into time geography models. As geographers and geographic information scientists sharpen our focus on issues of equity, public health, and climate change impacts as related to travel demand (Kar et al. 2022), there is an opportunity to revisit time geography with a lens toward increasing methodological flexibility that is needed for inclusion of diverse experiences and individual preferences.

In this paper, our goal is to outline a research agenda for a modern time geography, capable of more flexibly representing the relationships between space, time and access and able to be nuanced for inclusion of more diverse experiences with access and barriers. We relate this work to Hägerstrand (1970) early work on time geography and, as such, focus on how time geography enables quantification of constraints and access. The time geography framework we imagine is different from the classic time geography, in that it enables inclusion of more diverse data and highlights modern opportunities and costs to constraints and access such as technology and social inequality. The proposed framework moves away from a classic top-down model that considers a unified set of space–time constraints and universal rules for all individuals and toward a bottom-up approach that allows variation in access based on the individual characteristics and experiences of the focal person or community over time and space. To meet this goal, we present a conceptual framework that is adapted from movement research in GIScience (Geographic Information Science) that incorporates internal, external, and structural processes influencing the spatial pattern of individual movement at multiple scales. We then present a research road map and identify gaps in methods available for implementation of a modern time geography. We do not intend this to be the final word. Rather, we hope that this is the start of a conversation about how to continue to advance time geography through a collaborative research agenda across time geography, movement analysis, and GIScience.

## Time geography: background

Time geography was developed by Hägerstrand in mid 1960s to study human migration (Hägerstrand 1970). By incorporating both time and geographic context in the analysis of movement, theories and methods of time geography have been instrumental in the geographer’s effort to nuance hypotheses regarding access. Time geography constrains movement based on maximum speed given in a particular time period. Hägerstrand (1970) categorizes the temporal and spatial constraints on access into three categories: “capability constraints”, “coupling constraints”, and “authority constraints”. Capability constraints are those that limit the accessibility of the individuals because of physical or biological traits (e.g., age, disability) or available mobility tools such as transportation mode. Coupling constraints are those that restrict movement because of people’s requirements to be at certain locations at certain times for certain time periods to perform social functions or meet with others, for example for schooling, buying goods, accessing jobs, or to socialize. Often fixed daily schedules of services and jobs create major constraints on access in space and time. Authority constraints are those formed by social and cultural rules and often are controlled by social organizations or the government. These constraints may impact the affordability or availability of resources for different individuals or populations and hence impact their movement patterns.

Implemented through methods like the space–time prism and space–time paths (Miller 2005), time geography can be used to define a potential path area (PPA), or the spatial extent of movement in a given time as possible locations that moving individuals can reach or create bundles to meet with other people (Patterson and Farber 2015). Time geography’s approach to constraints has also been adapted to model accessibility and spatial interaction in both physical and virtual spaces (Couclelis and Getis 2000). As well, by combining patterns in individual PPA, time geography is used as an approach to defining and comparing community level movement and social interaction across urban areas (Lee and Miller 2019; Farber et al. 2013), and to tracing fine scale dynamic interaction between individuals in space and time through movement (Dodge et al. 2021). Additionally, probabilistic time geography models the probable movement given a set of conditions, spatial context, or behavior by incorporating a more probable speed or path (Ahearn et al. 2017; Long et al. 2014; Song et al. 2016; Song and Miller 2014; Winter and Yin 2010a, b). Probabilistic time geography provides an important lens for understanding access which can call along a gradient, rather than be discrete. Time geography has been further developed to incorporate the influence of the physical environment on movement and access (Benitez-Paez et al. 2021) constraining movement to physical infrastructure and availability of services as a form of capability and authority constraints (i.e., transit) (Lee and Miller 2018).

Relative to our goal of developing a research agenda for modern time geography, we note two interesting trends in the development of time geography. First, time geography has made considerable advancements in the inclusion of geographic context data in analysis, most of the geographic data included represents the physical or built environment, such as topography, road networks, or locations of services (Ahearn et al. 2017; Kuijpers et al. 2010). Social and cultural studies use theories of time geography, but less common are the application of quantitative methods like space time prisms to model experiences of different individuals in response to different forms of authority constraints. A notable exception is research on gender in the context of capability and coupling constraints, which has used time geography as a theoretical and methodological framework to quantify differences in how space use over time varies by gender (e.g., Kwan 1999, 2000). Yet, even within analysis of aggregate gender difference, the focus is on differences in the distances of travel by gender and how this can increase or decrease access to resources and opportunities. Individual-specific factors such as concerns about safety, or social barriers to public space are known to impact genders differently but we have not seen these types of issues included in time geography.

A second noteworthy development in time geography studies is that methods have enhanced the delineation of paths, allowing variability of travel speed or accommodation for virtual access, for instance, by making velocity modeling sensitive to variability across space and time (Miller and Bridwell 2008), or customizing the activity types along the space–time path to incorporate virtual accessibility via telecommunication and Internet (Shaw and Yu 2009). Varying speed across a physical landscape or creating links that are not bound by travel time is important for nuancing barriers to access. As well, time geography links travel to models of spatial processes and has advanced them beyond prediction of generalized random walks to include more realistic processes of movement (Long et al. 2014) and more realistic interpolation of travel pathways. Research on how to represent travel speed and spatial processes that govern realistic travel have set the stage for more holistically modeling that includes a broader range of factors that influence movement. Building on this, future models should advance the capacity of time geography by not only considering mechanistic movement capacities such as travel speed but also incorporating a range of individual-specific traits that may limit or enhance their access to resources and opportunities in space and time.

## Framework for modern time geography

**Fig. 1:**
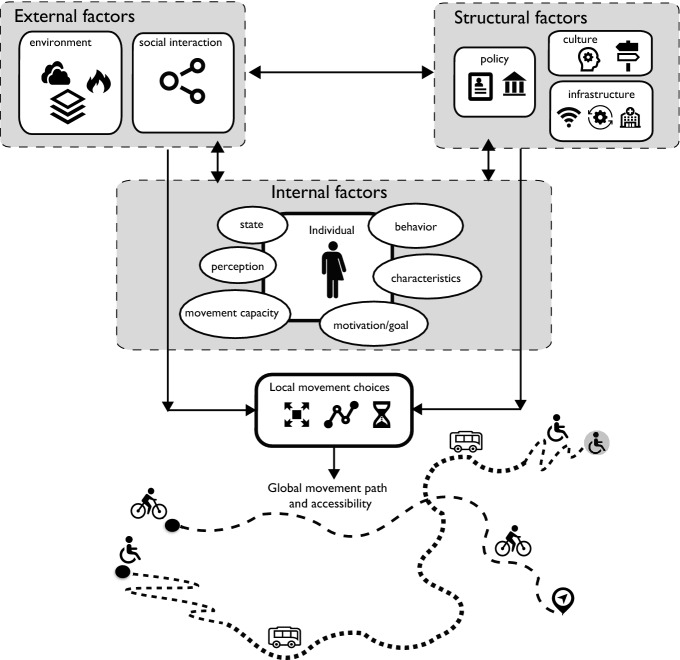
Framework for an individualized time geography representing varied access and movement choices based on influencing internal, external, and structural factors

Adapting existing conceptual frameworks for context-aware movement analytics that are offered mainly for movement ecology (Ahearn et al. 2017; Brum-Bastos et al. 2021; Nathan et al. 2008), we build a new framework to enhance time geography for modeling individualized access to resources and opportunities. Time geography constrains movement with time and speed. In contrast, with a less emphasis on time, the movement ecology paradigm (Nathan et al. 2008) describes organismal movement based on four basic mechanistic components: “the internal state (why move?), motion (how to move?), and navigation (when and where to move?) capacities of the individual and the external factors affecting movement”. Focusing on human movement, in our framework (illustrated in Fig. 1) we leverage the flexibility of the movement ecology paradigm to alleviate the physical and universal constraints of time geography for movement and accessibility in modern times. At the same time, we individualize time geography to differentiate access for individuals with diverse characteristics and how they experience a variety of *internal* (related to the individual), *external* (related to the environment), and *structural* (related to the community and cultural rules) factors. We further map the capability, coupling, and authority constraints introduced by Hägerstrand to these factors and discuss how the interplay between them influences the movement paths and accessibility of individuals in space and time. The interplay among these factors can directly or indirectly impact movement and drive or limit individuals’ access to resources at multiple scales—i.e., influencing their movement choices and interactions at local and individual scales and shaping their movement paths and access collectively at global and population scales.

### Individual level internal factors

Individuals’ behaviors and activities are driven by their needs and experiences which can vary significantly for each person depending on their physiological and biological characteristics, their personal capabilities and preferences as well as in space and time use. These individual level factors relate to Hägerstrand’s capability constraints on movement and accessibility. However, some internal factors might even be considered as the driver or enabler of movement and access (e.g., individual will, strengths, fitness level). Intentional movement historically has been used as a means to access resources, opportunities, and to conduct activities to satisfy one’s needs and goals. Movement often is initiated based on a change in the psychological state (e.g., craving, feeling tired, hungry, other mental state) of an individual or an urge to access resources. The change in psychological state then triggers a behavior which sets the movement goal (e.g., engaging in an activity, commuting, exercising, etc.). For example, we move to commute to work (the need to access a job, earn money, and social status), to get food when we are hungry or feel unsafe (change in internal state), and for vacation or recreation (the desire to see a new place or the need to exercise), or simply to explore or reach a place for a specific activity or need (shopping, dining, receiving health care, etc.). Other internal factors that influence movement and access are individuals’ inherent characteristics (behavioral traits, biological state, gender, age), perceptions (feeling safe or unsafe), and movement capacities (physiological characteristics, mobility, disability). Although these traits are associated with the focal individual, their impact on movement choices can be influenced by other external (e.g., environment, geography) and structural factors (e.g., policy, social organization) as described later. That is, the intersections of these individual level factors with the three forms of capability, coupling, and authority constraints may shape space–time paths of individuals differently. As a result, different people and populations may experience the same environments in totally different ways. There are many examples of literature that describe variability in access to a wide range of conditions and services including: transit (Lubitow et al. 2017), active transportation (Lee et al. 2017; Agyeman and Doran 2021). The interplay of individual-specific factors and other factors can generate different experiences, responses, and preferences for different people in their movement decisions and travel demands (Kar et al. 2022). As such, these factors may have a varied effect on people’s movement choices including their destinations, movement means and capacities, and time constraints, and hence differentiate how they access resources and opportunities (Brown 2022). For example, a woman might not feel safe to walk in unfamiliar or quiet locations at dark (environmental factor), because of the perceived risk of crime (structural issue).

### Local and global scale external factors

Movement is highly influenced by the external context within which it takes place. The built environment, available physical and cyber infrastructure, geography, and social interactions impact individuals’ route choices, schedules, transportation modes, destinations, and even their internal states, perceptions and movement capacities. As such, these factors can also impact the aforementioned individual level internal factors, and how they drive their movement choices. That is, the external factors might expose all individuals or be available to all similarly (e.g., road network, weather, location of hospitals) or their impact on access might vary for different individuals (e.g., perception of safety or risk by man or woman, affordance of various types of transportation, extreme weather events experienced by people with or without disability, varied health insurance providers). The influence of external factors may also vary at different scales. At the local scale, movement choices of individuals may be impacted by their immediate environment (e.g., weather condition, time of day), while their global space–time path to access resources might be shaped by the broader build environment, infrastructure, and distribution and availability of services such as transportation. For example, movement paths of individuals are restricted by external factors such as the road networks and physical barriers generating capability constraints, or the condition of the place or the surrounding environment of the individuals (e.g., perceived as safe or unsafe) (Friman et al. 2020). Also, weather conditions can impact human mobility in urban areas and their impact can vary depending on the individuals’ capabilities or other internal factors such as disability, age, etc. (Brum-Bastos et al. 2018; Sagl et al. 2011). In association with authority constraints and internal factors, these factors may also influence individuals’ perception and hence impact their movement choices and interactions with other individuals. For example, at night or in a new and unfamiliar environment, some individuals might feel unsafe in quiet and dark places and may prefer to take a private vehicle to quickly reach their destinations, but during the day and in downtown areas or urban green spaces people might enjoy taking their time to explore on foot or by bike and to interact socially. The perception of a safe or unsafe space can be shaped by confounding factors such as people’s internal characteristics and identity as well as their experiences or perceptions of the structural elements (i.e., higher crime rates in certain locations) as it relates to their environment (e.g., dark, quiet). Therefore, external factors may play a role in capability constraints or coupling constraints, although they also contribute to authority constraints. For example, the locations of national parks and weather conditions (i.e., external factors) impact visitors' decisions to visit or not. However, their access to national parks may be limited depending on operation hours and entrance fees which are controlled by the authorities (structural factors) and the environmental conditions (e.g., risk of wildfire, storm). The interplay of individual-level local movement choices and their interaction with the immediate and broader environment will eventually shape the collective mobility patterns of the population at the community level at global scales.

### Community level structural factors

Structural factors are those that can provide opportunity or limitations for individuals and hence influence their access and shape their experiences differently. These factors largely intersect with Hägerstrand’s authority constraints. For example, policies, controlled access to or costs of infrastructure, social programs, policing, public health policies, crime mitigation, funding, and zoning restrictions that are put in place can limit or expand individuals’ access to certain resources. Although the entire community is exposed to or encounters these structural elements, the experience can significantly differ for different population segments. For example, transportation as an infrastructure (external factor) might not be affordable or accessible (structural factor) to all people based on their socioeconomic status, gender, disability (internal factors), or simply because of limited hours of operations. These factors can restrict or empower individuals in their movement choices and access to schools, hospitals, and other services. For example, depending on the insurance network or school district, individuals’ options might be limited to access health care or education. Incentives for using electric vehicles, supply-chain issues, lockdowns, admission challenges for various service providers or opportunities can also have an impact on movement choices and individual’s access. Cultural differences might also drive individuals’ decisions in how and which resources are accessed, impacting public policies and funding for building infrastructure. Community level universal policies, such as the stay-at-home orders which were implemented to mitigate the COVID-19 pandemics, are an example of how policy can have different repercussions for different individuals and may result in inequitable access to resources and opportunities depending on individuals’ occupation type, income level, age, and gender (Long and Ren 2022).

### Space–time path and potential path area

The original space–time prism and potential path area model what portion of space is available to the individual while the space–time path represents the actual path of the individual over time (Miller 1991). In our framework, the space–time path and potential path area to access resources or perform activities are modeled on an individual level. First, the movement can be goal-oriented toward a destination or can be exploratory. The goal is determined by the person’s needs and intentions to access resources or to conduct an activity. Whether to move or not, the destinations, the bundles to interact with others, and the paths of movement are driven based on the interplay between the internal, external, and structural factors. For example, a person’s will to earn money moves them to go to work. Depending on the availability of the infrastructure and the policy, the person may access their job remotely or have to travel and meet with other people at certain locations or time. Access to job can also be facilitated on the go or at home via cyberinfrastructure. If there is a need to travel to work, the decision on how to travel is dependent on the accessibility and affordability of available public transportation (if any), weather conditions, as well as the person's capabilities, preferences and perception of different routes and means of transportation. As also pointed out in Hägerstrand (1970), people’s movement paths and the preferred locations to perform activities or find jobs, schools, services, etc. can also be impacted by their necessities, both physiological and physical, as well as cultural and policy rules. Our framework makes Hägerstrand time geography more flexible by allowing individuals’ preferences and choices to vary in space and in time through internal, external and structural factors, impacting individuals’ travel speed, destinations, and their time budget. As such, while the path decisions (origin, destination, departure, route choice) are made by the individual, the spatial and temporal constraints forming the shape of the potential path area are driven by the complex interplay of these factors, as illustrated in Fig. 1. We imagine that this can be modeled using a hyperspace rather than a three-dimensional space–time cube, in which each dimension models a specific factor. For example, perceptions and preferences of the individuals, environmental conditions, and availability and affordability of infrastructure are the different dimensions to the space, which contribute to a joint probability determining the locations accessible to the individual at a given time.

## Research roadmap for a modern time geography

**Fig. 2:**
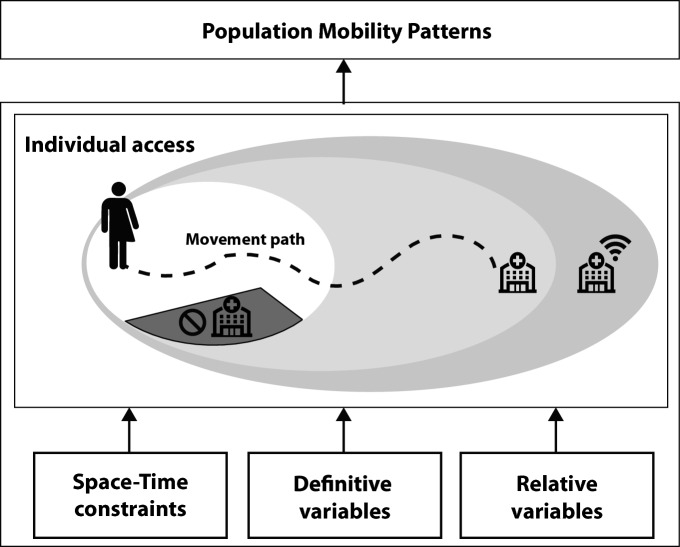
Roadmap for a research agenda in support of a modern time geography

Time geography has been a longstanding approach to modeling accessibility. In order for time geography to have the flexibility to support the proposed framework above and leverage the opportunities of big data, we need methods that implement nuanced individual experiences and address challenges of integrating big data at individual and population scales. We propose a roadmap for research that will enable us to continue to leverage time geography for studies of access (Fig. 2). Linking to the framework, we focus here on practicalities of implementing data driven time geography models and divide research needs into space–time constraints, definitive variables, relative variables, and the need to link between individual and population scales of analysis.

### Space–time constraints

A fundamental assumption of time geography is that travel time and coupling constraints determine what resources and opportunities people can reach over particular time frames. The internal, external, structural factors of the framework highlight that research is needed on how to implement constraints that are not governed by travel. Using the healthcare example, we highlight that healthcare can require travel to in person appointments or can be decoupled from travel time through e-health services. Further, health care workers may do the traveling providing in-home services, mobile health care centers may be utilized, and health care services can be provided by mail (i.e., at home COVID-19 test). Clearly, the spatial–temporal landscape that governs access to healthcare, and many other activities, is complex.

Hägerstrand’s theory of time geography accounted for digital access, including space–time paths of a phone call (Hägerstrand 1970), yet the implementation of broadly defined space–time constraints has proven more challenging. For nearly two decades, researchers have addressed the idea of access decoupled from travel time in time geography models. Janelle (2004) proposed typology for asynchronous and synchronous interactions, Shaw and Yu (2009) modified a space–time path to include interactions which happen digitally and presented a framework for hybrid physical–virtual space activities, and Couclelis (2009) argued for a revised time geography and a loosening links between activity, place, and time.

The acceleration of digital society has highlighted the need to also accelerate the flexibility of time geography models (Thulin et al. 2019). While there is research to build from, and we anticipate a boom of research on topics of how to include digital access in time geography models (Klapka et al. 2020; Shaw and Sui 2019), the tidy link between time constraints and access, which has stood the test of time, is currently being challenged. Yet, there are time geography implementations that are flexible and are already capable of integrating multiple constraints, including travel time and digital constraints. Bundling, or meshing space–time activities, is one example of a method that can combine travel paths across multiple travel models and can enable integration of non-travel constraints (Shaw and Yu 2009).

It is important to consider how change to time geography intersects more broadly with spatial analysis. Access and interaction decoupled from time also decouples from space and place. At a fundamental level the nature of spatial relationships or proximity is changing in response to society’s digitalization, inviting a revisit of Tobler’s first law of geography, which states that all things are related and near things more than far (Tobler 1970). The cyberspace and digital interaction challenge the way we have conceptualized Tobler’s law. The digitization of society, therefore, also requires a broadening of how we define near more generally in spatial analysis. Near may occur in physical space, but it may also occur in digital space. Research is needed on the theory and practice of integrating digital interaction in spatial weights matrices to allow representations of digital flow, interaction, and proximity in studies where nearness may best be represented digitally. In short, these issues are not the burden of time geographers, but require new models across geography.

While including non-time constraints in time geography models is not new, implementation is complex, and the problem has become combinatorial. The computational processing required to model access with complexity of multi-modal travel, digital access, and reliance on home deliveries presents a challenge. The computation complexity of time geography hinders modern research on accessibility using high frequency tracking and data streams as well as in real-time applications. Coupled with parallel processing and high-performance computing strategies, time geography can be enhanced for computing accessibility using large and heterogeneous space–time data. However, new methods are needed for careful handling of `time’ when using parallel processing in modern time geography (Dodge 2021). Simply breaking movement trajectories by slicing through time, as applied in spatial indexing approaches, might not be the best solution as it may alter movement speed and non-travel time. Also, as it is the case in the spatial dimension, the Modifiable Areal Unit Problem (MAUP) requires careful consideration when handling the time dimension in both physical and cyber spaces (Su et al. 2022). Hence, as with all modeling there remains a need to balance realism with complexity and to design models based on the need of research questions. In our framework we find it helpful to quantify access at an individual level, where access can be a combination of physical travel, digital interaction, and integration of other services.

### Modeling constraints through definitive variables

Following the proposed framework for modern time geography, we consider how inclusion of external, internal, and structural factors can allow more nuanced hypotheses on access to be addressed. In terms of a roadmap of research needs, we split external, internal, and structural factors into those that can be mapped *definitively* and those that are *relative*. Definitive variables are black and white, yes or no. Definitive variables include physical forms, as well as impermeable social structures and individual characteristics. Standard examples of constraints that can be modeled through definitive variables in time geography are transportation networks (Kuijpers et al. 2010) or location of services (Lee and Miller 2018). Definitive variables could also be used to represent structural factors, such as immigration policies of a nation or internal factors, such as a person’s mobility characteristics. The common aspect of definitive variables is that they can be quantified discretely.

A success of time geography implementation has been inclusion of definitive-type constraints that impact travel time, roads, residents, destinations. Though computationally there are limits, additional definitive variables are conceptually straightforward to include in time geography models. The primary challenge, from our perspective, will be accessing reliable data on digital networks and constraints. While the notion that maps of transportation infrastructure should be public and open is well established, it is not clear that the same approach is being taken to mapping digital infrastructure, though some companies are investing substantial resources into digital infrastructure mapping. Multi-scale maps of Wi-Fi and cell coverage are important for understanding digital access and must include where services exist as well as where services are and are not utilized. Open Street Maps is one platform that is well suited for mapping of digital infrastructure and its use, and would benefit from established typologies and organization of crowdsource mapping and consistent processing efforts to ensure consistency (Sehra et al. 2020). As well, there are apps that exist for personal mapping of cell and Wi-Fi coverage, however, if data are proprietary they lack broader benefit to the research community.

### Modeling constraints through relative variables

Relative constraints are external, internal, and structural factors that are not binary. They may be quantitative and continuous, categorial, or qualitative. Relative constraints can occur along a gradient and may or may not be “fuzzy”. Relative constraints could be a variable, like elevation, which varies continuously or could be a factor, like perception, which may be qualitative or include some level of subjectivity in the definition. These constraints can also be modeled through a combination of multiple variables (e.g., joint probability of multiple internal, external, and structural factors). In movement ecology, continuous variables (i.e., elevation and forest cover) are frequently used in modeling space utilization and movement (Ahearn et al. 2017), and many variables have some level of subjectivity or uncertainty (i.e., habitat suitability) because they are generated from other models. As wildlife cannot be asked directly about constraints and access, movement is the signal. While the same is true for human movement research, our understanding of systems creates more ability to use relative variables. Movement research in GIScience, which relies heavily on context through the inclusion of landscape and environmental variables, may be a guide for considering how to incorporate relative constraints in time geography models (Brum-Bastos et al. 2021).

As we sharpen our focus on social, economic, and demographic variation in access, there is a need to include more relative constraints in time geography models. There are examples of relative variables such as emotions being incorporated into space–time path (McQuoid and Dijst 2012) and these provide important foundations for accelerating these types of studies. In some cases, the limitation to including relative variables is simply lack of data. Definitive constraints like roads are much easier to map than relative constraints which could include perceptions or experience of using transportation mode, and which can also vary over time. Yet we know, that these subjective factors like perception matter. For example, perceptions of safety are considered the primary barrier to people using bicycles for transportation and disproportionately constrains women and inexperienced bicyclists (Winters et al. 2011). Crowdsourced data offer one approach to collecting more diverse and individual experience (Nelson et al. 2020), yet crowdsourcing lacks systematic sampling and can be biased toward people that have access to technology.

An interesting aspect of relative variables is that they will typically govern access on a gradient. Taking again the example of perceptions of bicycling safety, an individual will weigh the benefit of bicycling on a particular route against the potential cost. Value judgments that weight costs and benefits could include: how much faster will I arrive at my destination if I take a less safe route? How much do the physical health benefits of exercise outweigh potential risks of a crash? Methodologically, how we define the cost and benefit and weight these judgements against one another based on a wide range of individual factors is a challenge. Tools for cost benefit analysis are common in fields like economics (Bateman et al. 2003) and public health (Frew 2010), which are often implemented spatially, and provide a guide for how we can incorporate more diverse perspectives and may produce useful insights for advancing spatial methods.

### Individual versus population scales

Scale is often a challenge in spatial analysis and modeling, and as we move toward a more flexible time geography issues of scale will continue to require attention. Much of the big spatial data available on movement is broadly available only at the aggregate level. For good reason, protection of privacy, individual movements cannot be made widely available (e.g., Seidl et al. 2016). Rather, large volumes of individual trajectory data tend to be held by companies with proprietary data models. Yet, we know all big data are influenced by who is included and excluded from sampling, and that variation in the individual movements will be critical for understanding questions of equity and inclusion (Noi et al. 2021). How mobility and access of individuals in underrepresented groups compares with population level trends is critical, but impossible to quantify from aggregate data. There is no easy answer, but as a first step, GIScientists must be clear about who is and who is not represented in mobility data and time geography analysis.

Further, population movement data obtained via aggregate mobility indices provide a cross-sectional view of mobility. Future accessibility models should provide tools to integrate these data with the transitional view of individual movement choices and preferences over time. There is exciting potential to link individual data with aggregate level data to better understand variability in access and while the GIScientist is adept at linking data across scales, the details always require careful consideration. This is important for inclusion of diverse experiences and constraints on accessibility in modeling and prediction of population level mobility, for example, for more equitable crisis mitigation and resource allocation (Franklin et al. 2022).

### Understanding multiple process through data and analysis at multiple scales

Like all spatial analysis, time geography is an approach to understanding pattern and process. In the case of time geography, the link being made is between patterns and processes that reflect movement. While there are benefits to thinking of the components or categories that constrain movement (*internal, external, and structural*) the process of movement, and patterns that emerge through our actions and that are captured in data, are the result of the intersections between all the elements that enable and constrain movement. Hägerstrand’s original work included capability as a constraint, and we still like this term, but we think capability may also enable movement or drive movement differently for different individuals. Movement capability includes what an individual is able to do physically and physiologically, with all the other physical, digital, and policy elements that may create barriers and opportunities. While this idea is not new, the data that we have available to understand intersectionality of movement is more plentiful than ever before. Perhaps it is the availability of data and computational power to understand the diverse, nuanced, and complex forces that generate our daily, monthly, annual, and life movements at the scale of both individuals, communities, and even countries that is the truly emergent trend in geographic science, including time geography.

Similar to the interplay between components of constraints, access is governed by processes that occur across spatial and temporal scales. As discussed above, the ability to move, for example, by bicycle is the combined effect of zoning (i.e., a community level structural factor) (Nieuwenhuijsen 2018), the built environment (i.e., a local and global external factor) (Fischer et al. 2020), and people’s physical abilities or physiological comfort with bicycling (i.e., an individual or internal factor) (Branion-Calles et al. 2017). Each of these factors influence a person’s bicycling behavior at a particular scale, and creates ridership patterns that can also be measured at a variety of scales. The multiscale influence is not unique to time geography, rather it is at the core of all spatial analysis. Although, we are in an exciting time, as the availability of spatial and mobility data allow us to represent the range and interaction of spatial and temporal processes in our models, we still often struggle with methods that are truly multiscale, especially in time geography where multiscale may need to consider both spatial and temporal dimensions. Development of methods that allow us to differentiate the influence of various factors will help us to prioritize where to place investments in order to optimize access.

## Conclusion

Time geography is a theoretical framework that has stood the test of time, allowing researchers to better understand and quantify access. However, the accelerated digitalization of society combined with a focus on understanding how access varies across race, income, sexual identity, and physical limitations requires new consideration for how we include constraints in our studies of access. In this paper we present a more flexible framework for modern time geography to model individualized access by incorporating internal characteristics and varied impact of external and structural factors on individuals’ access to resources and opportunities. Research is needed to advance how time geography includes constraints decoupled from time, including digital access and delivery which provide access to diverse opportunities and services. While there is a body of work to draw from, mainstream approaches are needed to balance model complexity with realism. To support inclusion of digital access we need to address the lack of available mapping on digital infrastructure and develop new approaches to collect and utilize data that is relative, assessing access along a gradient, and compare individual and population level access. It is an exciting era for time geography, given the range of questions that benefit from the established approach to understanding access across scales. Yet, there are massive opportunities for all geographers to consider how digitization of life has changed the intersections of space, place, and time and to stretch our ability to understand diverse barriers to access at individual and population scales. Opportunities to model constraints and access in time geography are fueled by new mobility processes, growing availability of geospatial and mobility data and may benefit from interdisciplinary perspectives.
